# Early Response after Catheter Ablation of the Epicardial Substrate in a Patient with Brugada Syndrome Can Be Predicted by High Precordial Leads

**DOI:** 10.1155/2018/5980380

**Published:** 2018-04-29

**Authors:** Yae Min Park, Mi Sook Cha, Hanul Choi, Woong Chol Kang, Seung Hwan Han, In Suck Choi, Eak Kyun Shin, Young-Hoon Kim

**Affiliations:** ^1^Cardiology Division, Department of Internal Medicine, Gachon University Gil Medical Center, Incheon, Republic of Korea; ^2^Cardiology Division, Department of Internal Medicine, Korea University Anam Hospital, Seoul, Republic of Korea

## Abstract

A 52-year-old male with Brugada syndrome presented with repeated and appropriate shock from an implantable cardioverter defibrillator (ICD). Catheter ablation for substrate elimination targeting low-voltage, complex, and fractionated electrocardiograms and late potentials in the epicardial right ventricular outflow tract was successfully performed. Brugada phenotype in the right precordial leads from the third intercostal space disappeared in the early stage after catheter ablation and that from the standard fourth intercostal space disappeared later. He remained free from ventricular fibrillation over the next fourteen months. We suggest that this novel ablation strategy is effective in Brugada syndrome patients with ICD, and early response after catheter ablation can be predicted by high precordial leads.

## 1. Introduction

Brugada syndrome is characterized by a typical electrocardiogram (ECG) abnormality in the right precordial leads and a high risk of ventricular fibrillation- (VF-) related sudden cardiac death. The cornerstone to prevent sudden cardiac death is implantation of an implantable cardioverter defibrillator (ICD) in the high-risk patients [1]. Recently, a novel approach of substrate modification in the anterior epicardial region of the right ventricular outflow tract (RVOT) reduces VF episodes and even eliminates the Brugada phenotype in ECG [2]. The ST segment elevation in a Brugada syndrome patient is the most prominent in the leads overlying the RVOT and closely follows its anatomic position. Here, we present the case of successful substrate elimination in the epicardial RVOT in a patient with Brugada syndrome whose Brugada phenotype of high precordial leads in ECG disappeared in the early stage after catheter ablation.

## 2. Case Report

**Figure 1:**
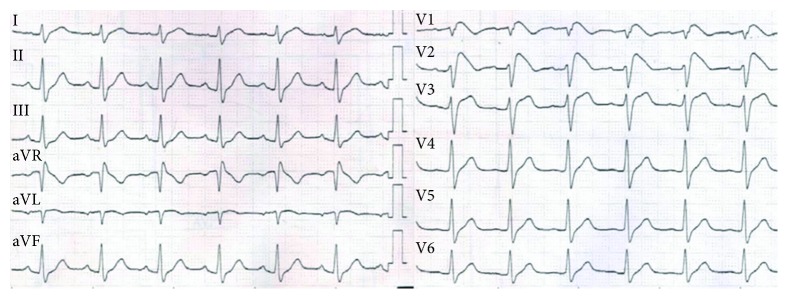
ECG after stabilization showed sinus rhythm with type I Brugada pattern, resulting in the diagnosis of Brugada syndrome. ECG = electrocardiogram.

A 52-year-old male presented with aborted sudden cardiac arrest and was referred to our electrophysiology department for further workup. The initial ECG at the emergency room revealed VF. Biphasic 200-Joule defibrillation restored sinus rhythm, and cardiopulmonary function recovered without neurologic sequelae. He suffered from several episodes of the agonal breathing pattern and generalized tonic-clonic movement prior to admission. The family history and past history including structural heart disease, syncope, or sudden cardiac death were unremarkable. His ECG after stabilization showed sinus rhythm with type I Brugada pattern (Figure 1). Echocardiography demonstrated no structural heart disease with normal left ventricular ejection fraction, and cardiac magnetic resonance imaging scan demonstrated normal biventricular function with no late gadolinium enhancement. Laboratory findings did not show any electrolyte abnormalities. He received ICD implantation for the secondary prevention of sudden cardiac death. Genetic studies were recommended, although declined by his family due to high cost.

Later, regular ECG exams showed consistently unprovoked type I Brugada pattern. Six months after the ICD implantation, he experienced appropriate shock due to VF. Medication with quinidine was started, and the dose was titrated up to 200 mg tid. However, he suffered from repeated appropriate shocks 10 months after starting quinidine. Premature ventricular complexes (PVCs) were rare during continuous ECG monitoring, and VF-triggering PVCs were absent. Therefore, he was offered radiofrequency catheter ablation for substrate elimination.

**Figure 2:**
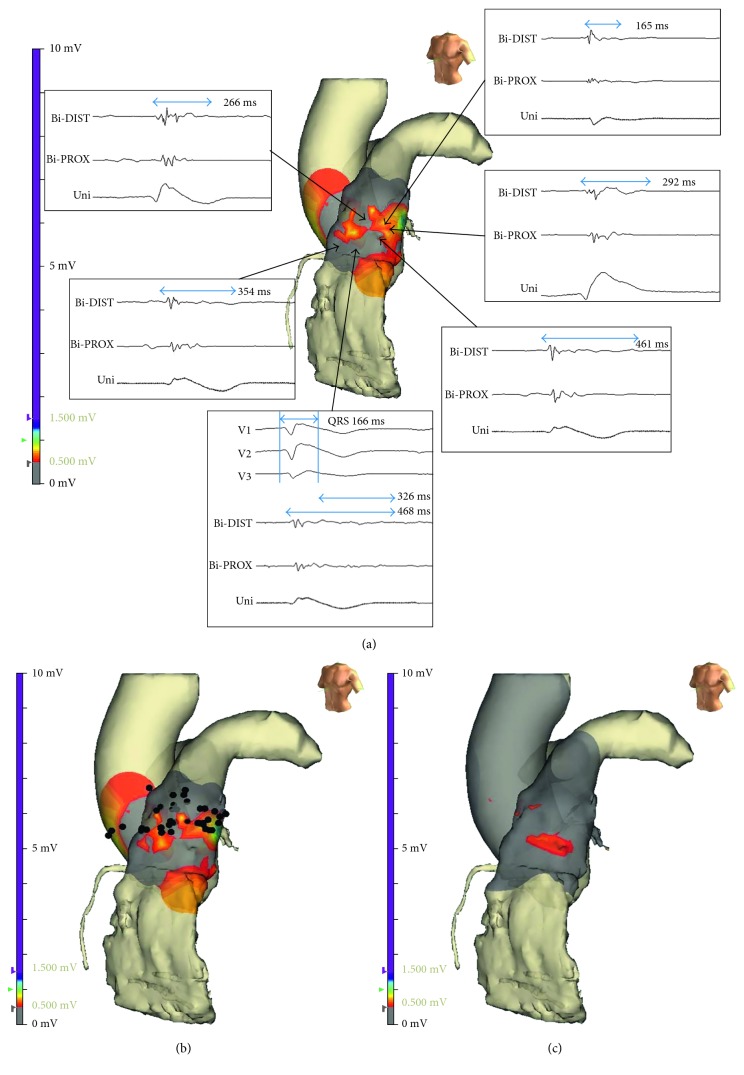
(a) Epicardial mapping revealed an extensive area of low voltage with fragmented and late potential in the anterior RVOT. Gray color of 3D mapping indicates scar tissue, and these areas were measured at 34.4 mm^2^, with the longest total duration of abnormal fractionated potential up to 468 ms and up to 326 ms after the end of the surface QRS. (b) Epicardial ablation was performed targeting the area of low voltage with fragmented and late potential in the anterior epicardial RVOT. (c) Remapped epicardial voltage mapping revealed that these areas were substituted with a scar. RVOT = right ventricular outflow tract.

In conscious sedation with intravenous propofol and midazolam, a VF induction test was attempted with programmed ventricular stimulation with three basal cycle lengths (600–500–400 ms) with up to three extra stimuli decremented to ventricular refractoriness or 200 ms from the RV apex prior to the ablation procedure, and VF was reproducibly inducible. Endocardial substrate mapping of the RV and RVOT was performed (NavX System; St. Jude Medical Inc., St. Paul, MN, USA), which showed no significant low-voltage area or fragmented potential. Epicardial access was obtained using fluoroscopy-guided subxiphoid puncture, and a long sheath (SL1; St. Jude Medical Inc., St. Paul, MN, USA) was introduced. Epicardial mapping (173 points) including RV and left ventricular areas was performed, revealing an extensive area of low voltage defined as bipolar signal amplitude <0.5 mV with wide duration (>80 ms) and fragmentation (≥3 distinct components) and late potential in the anterior RVOT. These areas were measured at 34.4 mm^2^, with the longest total duration of abnormal fractionated potential up to 468 ms and up to 326 ms after the end of the surface QRS. Unipolar mapping showed J-point elevation identical to the typical Brugada pattern (Figure 2(a)). Epicardial ablation was performed by an open irrigated tip catheter (Coolflex; St. Jude Medical, Minnetonka, MN, USA) targeting these areas (35 W; total ablation time: 19.8 minutes) (Figure 2(b)). This resulted in elimination of the fractionated potentials and drastic reduction of the local amplitude.

Remapped epicardial voltage mapping revealed that these areas were replaced with scar tissue (<0.1 mV; Figure 2(c)). Slight changes in morphologies of V1–V3 were detected, although the Brugada pattern remained in surface ECG right after the ablation procedure. An evaluation for the remnant substrate was performed after flecainide infusion (2 mg/kg for 10 minutes), revealing no significant changes in local amplitude in epicardial RVOT, although coved-type ST elevation in surface ECG was accentuated. The programmed ventricular stimulation was repeated, resulting in noninducibility of VF. The patient was discharged on the third day after the procedure with resumed quinidine therapy.

**Figure 3:**
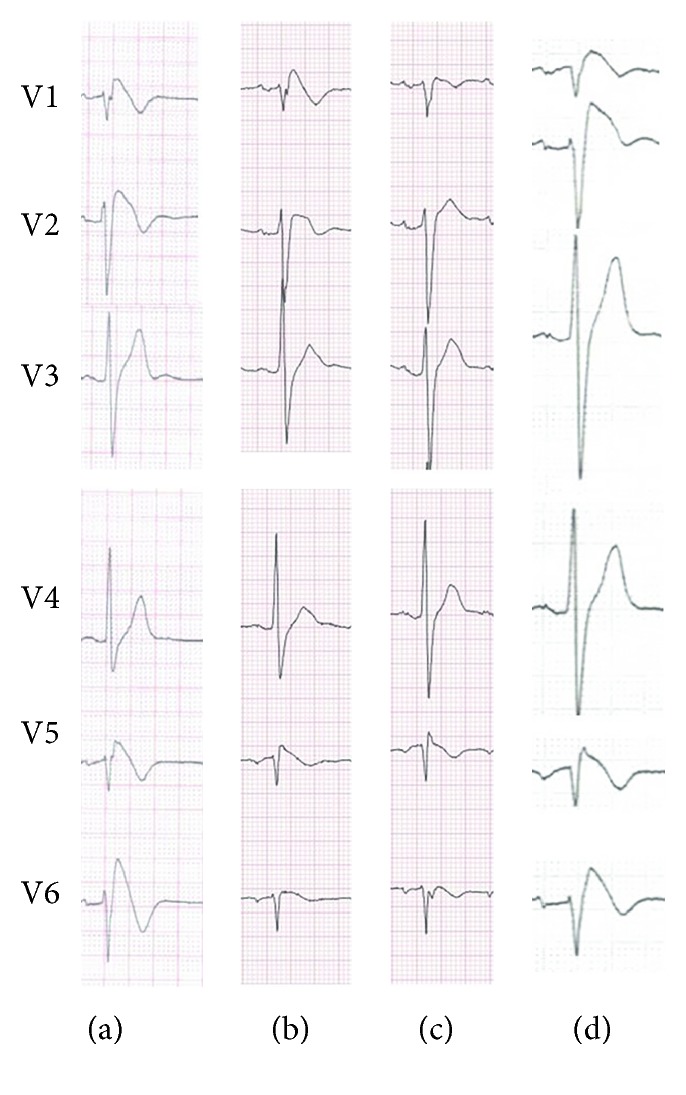
ECGs of the Brugada syndrome patient showing recordings of the anterior precordial leads: V1-2 leads from the 4th intercostal space and high precordial leads (V5 and V6 positioned cranially from V1 and V2) from the 3rd intercostal space. (a) Baseline before catheter ablation; (b) three days after ablation; (c) one month after ablation. Note the disappearance of the Brugada pattern at high precordial leads (V5-6) in the early stage and then at standard precordial V1-2 leads after catheter ablation. (d) The flecainide provocative test after three months of ablation revealed unmasking of type I Brugada pattern. ECG = electrocardiogram.

Right precordial leads from the third intercostal space showed disappearance of coved-type ST elevation on the third day after ablation, while those from the fourth intercostal space still showed the Brugada pattern. One month later, the Brugada pattern of precordial leads from either the third or fourth intercostal space disappeared (Figures 3(a)–3(c)). The flecainide provocative test (2 mg/kg for 10 minutes) was performed after three months of catheter ablation which revealed unmasking of type I Brugada pattern (Figure 3(d)). He remained free from palpitations, fainting, or syncopal episodes over the next fourteen months without quinidine treatment. There was no ventricular arrhythmia detected by the ICD.

## 3. Discussion

We report the case of Brugada syndrome patient with successful substrate elimination in the epicardial RVOT, who had been suffering from repeated appropriate ICD shocks. The Brugada phenotype in the high right precordial leads disappeared in the early stage after catheter ablation and that in the standard precordial leads disappeared later. Epicardial ablation in the anterior RVOT suppressed further VF episodes and completely eliminated the Brugada phenotype in this patient.

Catheter ablation for the management of Brugada syndrome has not been extensively reported. The epicardial ablation technique targeting the potential substrate in the RVOT for the Brugada syndrome was first introduced by Nademanee et al. in 2011 [3]. Delayed depolarization over the anterior aspect of the RVOT epicardium was suggested as the underlying electrophysiological mechanism in Brugada syndrome, and catheter ablation over this abnormal area resulted in normalization of the Brugada ECG pattern and prevented VF episodes [3]. A combined epicardial and endocardial ablation strategy for Brugada syndrome targeting low-voltage, fractionated late potentials in the RVOT was reported recently [4]. We performed endocardial substrate mapping before attempting the epicardial approach; however, there were no abnormal electrocardiograms to target.

The ST segment elevation in a Brugada syndrome patient is the most prominent in the leads overlying the RVOT and closely follows its anatomic position [5, 6]. Recording ECGs by placing the right precordial leads at higher intercostal spaces increases the sensitivity for detecting Brugada syndrome. Regional epicardial fibrosis and electrocardiogram abnormalities affecting the RVOT correspond to the high precordial leads where the Brugada phenotype is best seen and also an ablation effect is best seen in our patient.

We confirmed noninducibility of VF by programmed electric stimulation at the end of the procedure. However, noninducibility might not be the proper endpoint because of several shortcomings of the induction test. Recently, Pappone et al. published the catheter ablation of Brugada syndrome with endpoints of elimination of all abnormal electric ventricular potentials before and after ajmaline, leading to ECG normalization and noninducibility of VT/VF [7]. However, acute normalization of the Brugada ECG pattern during the ablation procedure needs further evaluation as an endpoint [8]. Despite normalization of spontaneous type I Brugada patterns after successful ablation, VF recurrence persisted in 27% of patients [9], and a substantial number of patients showed normalization of the Brugada pattern within three months of ablation [3]. Previous studies demonstrated that elimination of the arrhythmogenic substrate associated with disappearance of the Brugada ECG pattern as confirmed by flecainide testing indicates successful outcome [2, 7]. However, our patient showed unmasking of type I Brugada pattern by flecainide testing after three months of catheter ablation even though we ablated all abnormal substrates confirmed by the sodium channel blocker at the time of the ablation procedure. Currently, the best endpoint is to eliminate all substrate areas that harbor abnormal low-voltage fractionated signals detected after the sodium channel blocker challenge [8]. And we suggest that early disappearance of the Brugada pattern at high precordial leads is helpful to assess the success of ablation before disappearance of the Brugada pattern at standard precordial leads.

Epicardial ablation was effective in eliminating the substrate and the Brugada phenotype in our patient, and early successful response after catheter ablation can be predicted by high precordial leads.
